# Bottle-feeding practice and its associated factors among mothers of children aged 0 to 23 months in sub-Saharan Africa: a multi-level analysis of demographic and health surveys (2015–2022)

**DOI:** 10.1186/s12889-024-19244-9

**Published:** 2024-06-26

**Authors:** Enyew Getaneh Mekonen

**Affiliations:** Department of Surgical Nursing, School of Nursing, College of Medicine and Health Sciences, University of Gondar, Gondar, Ethiopia

**Keywords:** Bottle feeding, Young children, Sub-Saharan Africa, DHS, Multi-level analysis

## Abstract

**Background:**

Avoidance of bottle feeding is recommended as it interferes with optimal suckling behavior, is difficult to keep clean, and is an important route for the transmission of pathogens. However, there is a current shift towards breastfeeding for a short period and the introduction of bottle feeding in both the developed and developing worlds. Bottle-feeding practice and its individual- and community-level determinants are not addressed in sub-Saharan Africa. Therefore, this study aimed to fill this gap and assess the pooled prevalence and associated factors of bottle feeding among mothers of children less than 23 months of age.

**Methods:**

Data from the recent demographic and health surveys of 20 countries in sub-Saharan Africa conducted between 2015 and 2022 were used. A total weighted sample of 86,619 mother-child pairs was included in the current study. Multilevel mixed-effects logistic regression was used to determine the factors associated with the outcome variable. Intra-class correlation coefficient, likelihood ratio test, median odds ratio, and deviance (-2LLR) values were used for model comparison and fitness. Finally, variables with a p-value < 0.05 and an adjusted odds ratio with a 95% confidence interval were declared statistically significant.

**Results:**

The overall pooled prevalence of bottle feeding among mothers of children aged 0 to 23 months in sub-Saharan Africa was 13.74% (95% CI: 13.51%, 13.97%). Factors like maternal age [AOR = 1.09; 95% CI (1.04, 1.14)], educational status [AOR = 2.83; 95% CI (2.58, 3.10)], marital status [AOR = 1.16; 95% CI (1.09, 1.24)], maternal occupation [AOR = 0.76; 95% CI (0.73, 0.79)], media exposure [AOR = 0.80; 95% CI (0.76, 0.85)], wealth index [AOR = 1.21; 95% CI (1.15, 1.29)], sex of the household head [AOR = 1.17; 95% CI (1.12, 1.24)], family size [AOR = 1.06; 95% CI (1.01, 1.12)], number of under-five children [AOR = 1.11; 95% CI (1.04, 1.19)], place of delivery [AOR = 1.06; 95% CI (1.00, 1.12)], mode of delivery [AOR = 1.41; 95% CI (1.31, 1.52)], counseling on breastfeeding [AOR = 0.88; 95% CI (0.84, 0.92)], age of the child [AOR = 1.65; 95% CI (1.57, 1.75)], and residence [AOR = 1.64; 95% CI (1.56, 1.72)] were significantly associated with bottle-feeding practices.

**Conclusion:**

Nearly one out of seven children aged 0 to 23 months received bottle feeding in sub-Saharan African countries. Older mothers, higher mothers’ educational status, unmarried women, richest families, non-working mothers, exposed to media, female-headed households, large family size, having one under-five children, home delivery, cesarean delivery, children aged 6–11 months, and urban residence were significantly associated with an increased risk of bottle feeding. Breastfeeding promotion programs are advised to target mothers who are older, educated, working, rich, gave birth at home, have a large family size, are delivered by cesarean section, have children aged 6–11 months, and reside in urban areas to achieve a significant decrease in bottle feeding rates in sub-Saharan Africa.

**Supplementary Information:**

The online version contains supplementary material available at 10.1186/s12889-024-19244-9.

## Background

As per the “Convention on the Rights of the Child,” getting balanced nutrition is the right of every infant and child [1]. Appropriate infant and young child feeding (IYCF) practices among children aged 0–23 months are vital to improving their health, development, nutritional status, and survival [2]. Despite this, appropriate complementary feeding, dietary diversity, minimum feeding frequency, and age-appropriate feeding are achieved by only a few children [3]. Lack of optimal breast feeding and inappropriate complementary feeding practices are major causes of malnutrition, in which different countries worldwide are suffering from the double burden of both under and overnutrition [4]. Globally, around 45% of child mortality is associated with child undernutrition, and it is reported that 149 million, 45 million, and 37 million under-five children were estimated to be stunted, wasted, and overweight or obese in 2022, respectively [3].

Optimal breastfeeding of all children 0–23 months can save over 820,000 under-five children’s lives every year, contribute to higher income in adult life, and improve intelligence quotient and school attendance [5, 6]. However, the prevalence of exclusive breastfeeding among infants aged 0–6 months over the period of 2015–2020 was only 44% worldwide [7]. Bottle feeding is a form of infant feeding, either with breastmilk or formula, that has been used over the years [8]. Evidence showed that bottle-feeding was a main factor in child hospitalization due to infections, morbidity, and mortality [9–11]. It is also associated with rapid weight gain during infancy, which increases the risk of being overweight later in life [12]. The risk of dental caries is also higher among bottle-fed children than breast-fed children, which has a protective effect in early childhood [13]. Bottle-feeding has also had a negative impact on mothers, in which mothers who bottle-fed their children experienced negative emotions like guilt, anger, apprehension, uncertainty, and a sense of failure [14].

The World Health Organization (WHO) recommends avoiding bottle feeding as it may interfere with optimal suckling behavior, be difficult to keep clean, and be an important route for the transmission of pathogens [15]. In contrast to this recommendation, there is a current shift towards breastfeeding for a short period and the introduction of bottle-feeding in both the developed and developing worlds [16, 17]. Studies conducted elsewhere showed that the prevalence of bottle feeding was 37.9% in Indonesia [18], 39.7% in Sudan [19], 13.19% in 29 sub-Saharan African countries [20], 35.7% in Namibia [21], 12% in Ghana [22], 13.5% in Ethiopia [23], 42.7% in Woldia, Ethiopia [24], 35.5% in Asella town, Ethiopia [25], 35.0% in Agaro town, Ethiopia [26], and 19.6% in Holeta town, Ethiopia [27]. Different studies conducted in various countries also showed that age of the child [18, 23, 24, 27], sex of the child [18], maternal age [24, 27], educational status of the mother [18, 23], maternal occupation [18, 24, 25, 27], wealth index [18, 23], mode of delivery [18], residence [18, 19, 23], counseling on breastfeeding [19, 27], postnatal care (PNC) attendance [25, 27], number of under-five children [27], and place of delivery [25] were significantly associated with bottle feeding practice.

Bottle feeding was an optional indicator in the 2008 indicators for assessing IYCF practices, while the 2021 guideline declared that it is no longer an optional indicator and is recommended to be used in assessing feeding practices [2]. Bottle-feeding practice and its individual- and community-level determinants are not addressed in sub-Saharan Africa (SSA). Therefore, this study aimed to fill this gap and assess the prevalence and associated factors of bottle feeding among mothers of children less than 23 months of age using recent demographic and health surveys (DHS) (2015–2022) and indicators for assessing IYCF practices.

## Methods and materials

### Data sources, study design, and sampling

A cross-sectional pooled dataset using the recent DHS data from 20 SSA countries, which was conducted between 2015 and 2022, was employed. Demographic and health surveys from 20 SSA countries, including Angola (2015-16), Benin (2017-18), Burundi (2016-17), Ethiopia (2016), Gabon (2019-21), Gambia (2019-20), Guinea (2018), Kenya (2022), Liberia (2019-20), Mali (2018), Malawi (2015-16), Nigeria (2018), Rwanda (2019-20), Sierra Leone (2019), Senegal (2019), Tanzania (2022), Uganda (2016), South Africa (2016), Zambia (2018), and Zimbabwe (2015), were used. The data were appended to figure out the pooled prevalence of bottle feeding and its associated factors in SSA countries. Different datasets, including those for children, males, women, births, and households, are included in the survey for each country. For this study, the kid’s record (KR) file was used. The DHS is a nationwide survey, mostly collected every five years across low and middle-income countries. It makes cross-country comparison possible as it uses standard procedures for sampling, questionnaires, data collection, cleaning, coding, and analysis [28]. A total weighted sample of 86,619 mother-child pairs was included in the current study (Table 1). The DHS employs a stratified, two-stage sampling technique [29]. The first stage involves the development of a sampling frame, consisting of a list of primary sampling units (PSUs) or enumeration areas (EAs), which covers the entire country and is usually developed from the latest available national census. The second stage is the systematic sampling of households listed in each cluster, or EA. Further information on the survey sampling strategies is available in the DHS guideline [30].

**Table 1:** Sample size for prevalence and associated factors of bottle feeding among mothers of children aged 0–23 months in sub-saharan African countries

Country	Year of survey	Weighted sample (*n*)	Weighted sample (%)
Angola	2015-16	5,913	6.83
Benin	2017-18	5,479	6.33
Burundi	2016-17	5,276	6.09
Ethiopia	2016	4,078	4.71
Gabon	2019-21	2,593	2.99
Gambia	2019-20	3,433	3.96
Guinea	2018	2,987	3.45
Kenya	2022	4,045	4.67
Liberia	2019-20	2,224	2.57
Mali	2018	3,927	4.53
Malawi	2015-16	6,624	7.65
Nigeria	2018	12,513	14.45
Rwanda	2019-20	3,158	3.65
Sierra Leone	2019	3,856	4.45
Senegal	2019	2,485	2.87
Tanzania	2022	4,357	5.03
Uganda	2016	6,031	6.96
South Africa	2016	1,354	1.56
Zambia	2018	3,941	4.55
Zimbabwe	2015	2,345	2.71
Total sample size		86,619	100.00

### Variables of the study

#### Dependent variable

The outcome variable of this study was bottle feeding, which is defined as the proportion of children 0–23 months of age who were fed from a bottle with a nipple during the previous day [2]. Women who fed their children from a bottle with a nipple during the 24 h preceding the survey were considered to have practiced bottle feeding (“yes = 1”), while those who didn’t feed their children from a bottle with a nipple a nipple were considered not to have practiced bottle feeding (“no = 0”).

#### Independent variables

Both individual and community-level variables were considered to accommodate the hierarchical nature of DHS data. Individual-level variables: maternal age (15–24 years, 25–34 years, 35–49 years), educational status of mothers (no education, primary, secondary, higher), current marital status of the mother (unmarried, married), maternal occupation (not working, working), media exposure (no, yes), household wealth index (poor, middle, rich), sex of the household head (male, female), family size (1–4, 5–10, 11 and above), number of under five children (none, one, two, three and above), PNC checkup (no, yes), pregnancy intention (intended, unintended), place of delivery (home, health facility), mode of delivery (vagina, cesarean section), counseling on breastfeeding (no, yes), age of the child (0–5 months, 6–11 months, 12–23 months), sex of the child (male, female). Community-level variables: place of residence (urban, rural), community-level media exposure (low, high), community-level education (low, high), and community poverty level (low, high). These variables were created by combining individual-level variables, as these variables were not directly available from DHS data.

#### Descriptions of independent variables

**Media exposure** is generated by combining whether a respondent reads newspapers or magazines, listens to the radio, or watches television, and is coded as “yes” if the mother was exposed to at least one of these media and “no” otherwise.

##### Pregnancy intention

re-categorized as intended (if the pregnancy was wanted) and unintended (incorporating both mistimed and unintended).

##### Community-level of media exposure

the proportion of women who had been exposed to at least one media (television, radio, or newspaper) and categorized based on the national median value as low (communities with ≤ 50% of women exposed) and high (communities with > 50% of women exposed).

**Community-level education**: the proportion of women with a minimum primary level of education derived from data on respondents’ level of education. Then, it was categorized using the national median value into two categories: low (communities with ≤ 50% of women having at least primary education) and high (communities with > 50% of women having at least primary education).

##### Community poverty level

an aggregated variable from household wealth status (proportion of women from poor and rich wealth status), and it was recoded as low and high community poverty level, likewise.

### Data management and analysis

Data extracted from the recent DHS data sets were cleaned, recoded, and analyzed using STATA/SE version 14.0 statistical software. Sample weight was employed to manage sampling errors and non-responses. Continuous variables were categorized, and categorical variables were further re-categorized. Descriptive analysis was carried out to present the data in frequencies and percentages. Both the individual and community-level variables were presented using descriptive statistics. The DHS data’s variables were organized in clusters; 86,619 mother-child pairs are nested within households, and households were nested within 1692 clusters. The assumptions of independent observations and equal variance across clusters were broken to employ the traditional logistic regression model. This is an indication that using a sophisticated model to take into account between-cluster factors is necessary. As a result, multilevel mixed-effects logistic regression was used to determine the factors associated with bottle feeding. Multilevel mixed effect logistic regression follows four models: the null model (outcome variable only), mode I (only individual-level variables), model II (only community-level variables), and model III (both individual and community-level variables). The model without independent variables (the null model) was used to check the variability of bottle feeding across the cluster. The association of individual-level variables with the outcome variable (Model I) and the association of community-level variables with the outcome variable (Model II) were assessed. In the final model (Model III), the association of both individual and community-level variables was fitted simultaneously with the outcome variable. The magnitude of the clustering effect and the degree to which community-level factors explain the unexplained variance of the null model were quantified by checking the intra-class correlation coefficient (ICC) and proportional change in variance (PCV). A model with the lowest deviance was selected as the best-fitted model. Finally, variables with a p-value less than 0.05 and an adjusted odds ratio (AOR) with a 95% confidence interval (CI) were described as statistically significant variables associated with bottle feeding. The presence of multi-collinearity between covariates was checked by using a variance inflation factor (VIF) falling within acceptable limits of 1–10, indicating the absence of significant collinearity across independent variables (supplementary file).

### Random-effect results

Random effects or measures of variation of the outcome variable were estimated using the median odds ratio (MOR), ICC, and PCV. The variation between clusters was measured by the ICC and PCV. Taking clusters as a random variable, the ICC reveals that the variation of adequate PNC between clusters is computed as ICC = VC/ (VC + 3.29) ×100%. The MOR is the median value of the odds ratio between the area of the highest risk and the area of the lowest risk for bottle feeding when two clusters are randomly selected, using clusters as a random variable; MOR = 𝑒 ^0.95√VC^. In addition, the PCV demonstrates the variation in the prevalence of bottle feeding explained by factors and computed as; PCV = (Vnull-VC)/Vnull×100%; where Vnull = variance of the null model and VC = cluster level variance [31]. The fixed effects were used to estimate the association between the likelihood of bottle feeding and individual and community-level independent variables.

## Results

### Individual- and community-level characteristics of mother-child pairs

A total of 86,619 mother-child pairs took part in this study. The mean age of mothers was 27.83 ± 0.02 years, and 45.49% of them fall in the age range of 25–34 years. More than one-third (35.49%) of mothers had no formal education, and 86.11% of them were married. More than two-thirds (69.28%) of mothers had jobs, and 63.45% of them had media exposure. More than three-fourths (78.44%) of mothers were from male-headed households, and 47.78% of them had poor economic status. More than half (58.89%) of mothers had 5–10 family members, and 40.52% of them had two under-five children in the household. More than two-thirds (67.43%) of mothers had no PNC checkup, and 69.99% of them had an unintended pregnancy. The majority (93.83%) of mothers had vaginal delivery, and 71.98% of them gave birth at a health facility. More than half (52.63%) of mothers were not counseled on breastfeeding, and 68.44% of them were from rural areas. The mean age of children was 11.19 ± 0.02 months, and 48.76% of them were aged 12–23 months. Regarding the sex of the child, more than half (50.73%) of them were male. More than half (53.88%), 57.97%, and 54.74% of mothers had low community-level media exposure, low community-level education, and high community poverty levels, respectively (Table 2).

**Table 2:** Individual-and community-level characteristics of mother-child pairs, pooled data from 20 SSA countries, DHS 2015–2022

Variables	Category	Frequency (*n*)	Percentage (%)
Maternal age	15–24 years	30,809	35.57
	25–34 years	39,402	45.49
	35–49 years	16,408	18.94
Educational status of mothers	No education	30,745	35.49
	Primary	28,859	33.32
	Secondary	23,285	26.88
	Higher	3,730	4.31
Current marital status of the mother	Unmarried	12,030	13.89
	Married	74,589	86.11
Maternal occupation	Not working	26,608	30.72
	Working	60,011	69.28
Media exposure	No	31,663	36.55
	Yes	54,956	63.45
Household wealth index	Poor	41,391	47.78
	Middle	17,115	19.76
	Rich	28,113	32.46
Sex of the household head	Male	67,942	78.44
	Female	18,677	21.56
Family size	1–4	23,993	27.70
	5–10	51,008	58.89
	11 and above	11,618	13.41
Number of under five children	None	1,673	1.93
	One	26,737	30.87
	Two	35,099	40.52
	Three and above	23,110	26.68
PNC checkup	No	56,241	67.43
	Yes	27,164	32.57
Pregnancy intention	Intended	25,995	30.01
	Unintended	60,617	69.99
Place of delivery	Home	24,269	28.02
	Health facility	62,350	71.98
Mode of delivery	Vaginal	81,113	93.83
	Cesarean section	5,337	6.17
Counseling on breastfeeding	No	43,981	52.63
	Yes	39,586	47.37
Age of the child	0–5 months	22,678	26.18
	6–11 months	21,704	25.06
	12–23 months	42,237	48.76
Sex of the child	Male	43,938	50.73
	Female	42,681	49.27
Place of residence	Urban	27,341	31.56
	Rural	59,278	68.44
Community-level media exposure	Low	46,668	53.88
	High	39,951	46.12
Community-level education	low	50,211	57.97
	High	36,408	42.03
Community poverty level	Low	39,204	45.26
	High	47,415	54.74

### Pooled prevalence of bottle feeding among mothers of children aged 0 to 23 months

**Fig. 1:**
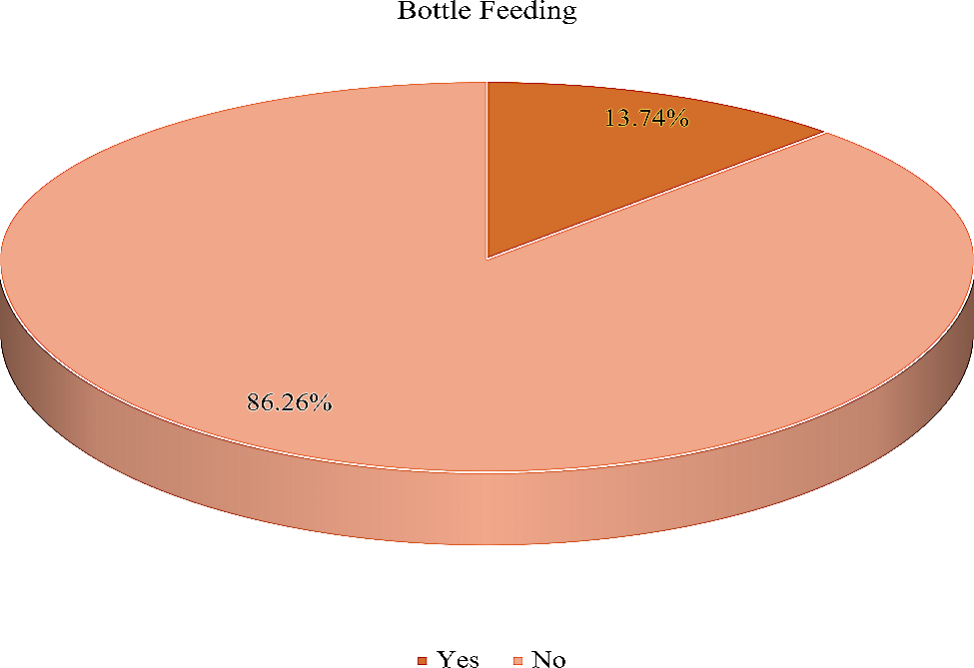
Pooled prevalence of bottle feeding among mothers of children aged 0 to 23 months in sub-Saharan African countries; DHS 2015–2022 (*n* = 86,619)

**Fig. 2:**
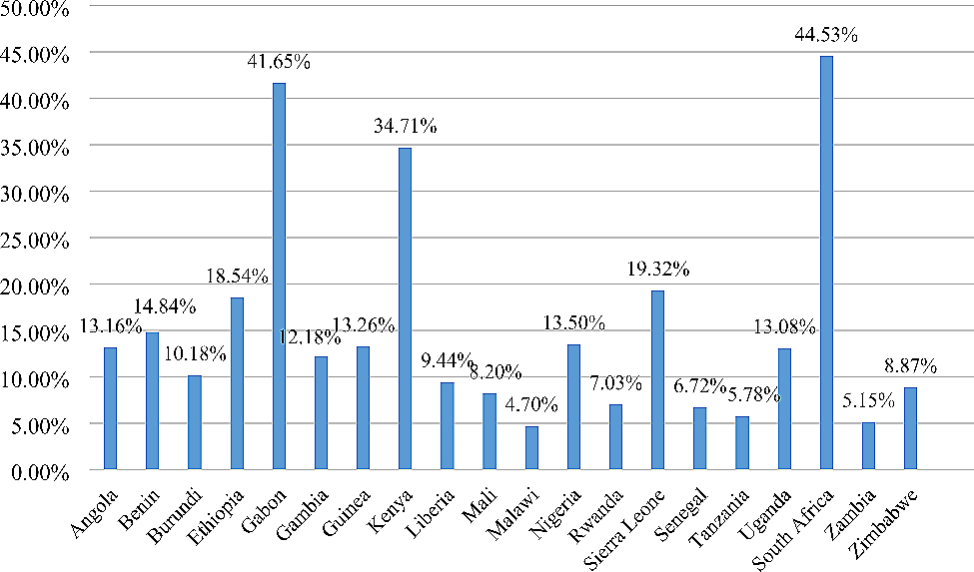
Prevalence of bottle feeding by country among mothers of children aged 0 to 23 months in sub-Saharan African countries; DHS 2015–2022 (*n* = 86,619)

The overall pooled prevalence of bottle feeding among mothers of children aged 0 to 23 months in sub-Sharan African countries was 13.74% (95% CI: 13.51%, 13.97%) (Fig. 1). The highest prevalence of bottle feeding was reported in South Africa (44.53%), and the lowest in Malawi (4.70%) (Fig. 2).

### Measures of variation and model fitness

A null model was used to determine whether the data supported the decision to assess randomness at the community level. Findings from the null model showed that there were significant differences in bottle feeding between communities, with a variance of 0.1547566 and a P value of < 0.001. The variance within clusters contributed 95.51% of the variation in bottle feeding, while the variance across clusters was responsible for 4.49% of the variation. In the null model, the odds of bottle feeding differed between higher- and lower-risk clusters by a factor of 1.45 times. The intra-class correlation value for Model I indicated that 2.11% of the variation in bottle feeding accounts for the disparities between communities. Then, with the null model, community-level variables were used to generate Model II. According to the ICC value from Model II, cluster variations were the basis for 3.49% of the differences in bottle feeding. In the final model (model III), which attributed approximately 2.15% of the variation in the likelihood of bottle feeding to both individual and community-level variables, the likelihood of bottle feeding varied by 1.28 times across low and high bottle feeding (Table 3).

**Table 3:** Model comparison and random effect analysis for bottle feeding and its associated factors in SSA countries, DHS 2015–2022 (*n* = 86,619)

Parameter	Null model	Model I	Model II	Model III
Variance	0.1547566	0.0709353	0.1188767	0.0723905
ICC	4.49%	2.11%	3.49%	2.15%
MOR	1.45	1.25	1.39	1.28
PCV	Reference	54.16%	23.18%	53.22%
Model fitness				
LLR	-34483.957	-31330.965	-33396.336	-31097.996
Deviance	68,967.914	62,661.93	66,792.672	62,195.992

*ICC* Intra cluster correlation, *LLR* log-likelihood ratio, *MOR* median odds ratio, *PCV* Proportional change in variance

### A multi-level analysis of factors associated with bottle-feeding

In the final fitted model (model III) of multivariable multilevel logistic regression, maternal age, educational status of mothers, current marital status, maternal occupation, media exposure, wealth index, sex of the household head, family size, number of under five children in the household, place of delivery, mode of delivery, counseling on breastfeeding, age of the child, residence, community-level media exposure, and community-level education were significantly associated with bottle feeding among mothers of children aged 0 to 23 months in SSA countries.

The odds of bottle feeding were 1.09 and 1.08 times higher among mothers aged 25–34 years and 35–49 years compared with those aged 15–24 years, respectively [AOR = 1.09; 95% CI (1.04, 1.14)] and [AOR = 1.08; 95% CI (1.02, 1.16)]. Mothers of children who completed secondary and higher education were 1.61 and 2.83 times more likely to practice bottle feeding compared with those who had no education, respectively [AOR = 1.61; 95% CI (1.51, 1.71)] and [AOR = 2.83; 95% CI (2.58, 3.10)]. Unmarried women were 1.16 times more likely to practice bottle-feeding than married women [AOR = 1.16; 95% CI (1.09, 1.24)]. Non-working mothers were 24% more likely to practice bottle feeding compared with their counterparts [AOR = 0.76; 95% CI (0.73, 0.79)]. Mothers with media exposure were 20% more likely to practice bottle feeding compared with those who were not exposed to media [AOR = 0.80; 95% CI (0.76, 0.85)]. Mothers from rich households were 1.21 times more likely to practice bottle feeding compared with those from poor households [AOR = 1.21; 95% CI (1.15, 1.29)].

Likewise, female-headed households were 1.17 times more likely to practice bottle-feeding compared with male-headed households [AOR = 1.17; 95% CI (1.12, 1.24)]. The odds of bottle feeding were 1.06 times higher among mothers with 5–10 household members compared with those with 1–4 household members [AOR = 1.06; 95% CI (1.01, 1.12)]. Mothers with one under-five child in the household were 1.11 times more likely to practice bottle feeding compared with those who had three or more under-five children [AOR = 1.11; 95% CI (1.04, 1.19)]. Mothers of children who gave birth at home were 1.06 times more likely to practice bottle feeding compared with those who gave birth at a health facility [AOR = 1.06; 95% CI (1.00, 1.12)]. Cesarean section delivery also increases the odds of bottle feeding by 1.41 times [AOR = 1.41; 95% CI (1.31, 1.52)]. Mothers who were counseled on breastfeeding were 12% more likely to practice bottle feeding compared with their counterparts [AOR = 0.88; 95% CI (0.84, 0.92)]. Children aged 6–11 months were 1.65 times more likely to receive bottle feeding compared with those aged 0–5 months [AOR = 1.65; 95% CI (1.57, 1.75)]. Mothers from urban areas were 1.64 times more likely to practice bottle feeding compared with those from rural areas [AOR = 1.64; 95% CI (1.56, 1.72)]. Mothers from communities with high media exposure were 1.06 times more likely to practice bottle feeding compared with those from low media exposure [AOR = 1.06; 95% CI (1.00, 1.13)]. Furthermore, mothers from communities with high education were 1.11 times more likely to practice bottle feeding compared with their counterparts [AOR = 1.11; 95% CI (1.05, 1.18)] (Table 4).

**Table 4:** Multivariable multilevel logistic regression analysis of individual and community-level factors associated with bottle feeding among women in SSA countries, DHS 2015–2022

Variables	Category	Model I AOR (95% CI)	Model II AOR (95% CI)	Model III AOR (95% CI)
Maternal age	15–24 years	1		1
	25–34 years	1.10 (1.06,1.17)*		1.09 (1.04,1.14)*
	35–49 years	1.11 (1.04,1.18)*		1.08 (1.02,1.16)*
Educational status of mothers	No education	1		1
	Primary	0.84 (0.79,0.89)*		0.84 (0.79,0.89)*
	Secondary	1.70 (1.60,1.81)*		1.61 (1.51,1.71)*
	Higher	3.09 (2.82,3.39)*		2.83 (2.58,3.10)*
Current marital status	Unmarried	1.20 (1.12,1.28)*		1.16 (1.09,1.24)*
	Married	1		1
Maternal occupation	Not working	1		1
	Working	0.74 (0.71,0.77)*		0.76 (0.73,0.79)*
Media exposure	No	0.77 (0.73,0.81)*		0.80(0.76,0.85)*
	Yes	1		1
Wealth index	Poor	1		1
	Middle	1.05 (0.99, 1.12)		0.97 (0.91,1.03)
	Rich	1.53 (1.45,1.61)*		1.21 (1.15,1.29)*
Sex of the household head	Male	1		1
	Female	1.19 (1.13,1.26)*		1.17 (1.12,1.24)*
Family size	1–4	1		1
	5–10	1.04 (0.99, 1.10)		1.06 (1.01,1.12)*
	11 and above	0.95 (0.87, 1.03)		0.96 (0.88,1.05)
Number of under five children	None	0.99 (0.86, 1.15)		1.01 (0.87,1.17)
	One	1.10 (1.03,1.18)*		1.11(1.04,1.19)*
	Two	0.96 (0.91, 1.02)		0.97 (0.91,1.03)
	Three and above	1		1
PNC checkup	No	0.96 (0.92, 1.01)		0.98 (0.93,1.02)
	Yes	1		1
Pregnancy intention	Intended	1		1
	Unintended	0.96 (0.92, 1.01)		0.97 (0.92,1.01)
Place of delivery	Home	1.04 (0.99, 1.10)		1.06 (1.00,1.12)*
	Health facility	1		1
Mode of delivery	Vagina	1		1
	Cesarean section	1.41 (1.31,1.52)*		1.41 (1.31,1.52)*
Counseling on breastfeeding	No	0.88 (0.84,0.92)*		0.88 (0.84,0.92)*
	Yes	1		1
Age of the child	0–5 months	1		1
	6–11 months	1.65 (1.56,1.74)*		1.65 (1.57,1.75)*
	12–23 months	0.97 (0.92, 1.02)		0.96 (0.92,1.02)
Sex of the child	Male	1		1
	Female	0.99 (0.95, 1.03)		0.99 (0.95,1.03)
Place of residence	Urban		2.56 (2.45,2.67)*	1.64 (1.56,1.72)*
	Rural		1	1
Community-level media exposure	Low		1	1
	High		1.20 (1.13,1.29)*	1.06 (1.00,1.13)*
Community-level education	low		1	1
	High		1.20 (1.12,1.28)*	1.11 (1.05,1.18)*
Community poverty level	Low		0.98 (0.92, 1.05)	0.96 (0.90,1.02)
	High		1	1

## Discussion

The present study was conducted to determine the pooled prevalence and associated factors of bottle feeding among mothers of children aged 0 to 23 months in sub-Saharan African countries using recent demographic and health surveys and IYCF indicators. The study revealed that the overall pooled prevalence of bottle feeding was 13.74% (95% CI: 13.51%, 13.97%). This finding was higher than studies conducted in Ethiopia (13.5%) [23], Ghana (12%) [22], and 29 sub-Saharan African countries (13.19%) [20]. On the other hand, this finding was lower than studies conducted in Woldia, Ethiopia (42.7%) [24], Asella town, Ethiopia (35.5%) [25], Agaro town, Ethiopia (35.0%) [26], Holeta town, Ethiopia (19.6%) [27], Indonesia (37.9%) [18], Sudan (39.7%) [19], and Namibia (35.7%) [21]. The plausible justification for this discrepancy might be due to differences in study area, sample size, socio-economic status, perception and knowledge of mothers towards optimal child and infant feeding, and data type. Some of the previous studies were conducted in a single study area with a small sample size, whereas the current study used pooled data from 20 SSA countries. Some studies also used primary data to estimate the prevalence of bottle feeding, while this study used nationally representative secondary data from recent DHSs in 20 countries.

This study also identified individual- and community-level variables significantly associated with bottle feeding. Accordingly, mothers aged 25–34 and 35–49 years had higher odds of bottle-feeding compared with those aged 15–24 years. This finding was inconsistent with studies conducted in Woldia, Ethiopia [24], and Holeta town, Ethiopia [27], in which the lower age of mothers was positively associated with bottle feeding. The higher odds of bottle-feeding among older women in this study might be due to the fact that mothers aged ≥ 25 years are more experienced and feel their babies are not getting enough breast milk. Those mothers are also more likely to have jobs and start back to work, which pushes them toward breastfeeding cessation and a shift to bottle feeding. This implies that nutrition interventions should consider older women to reduce the prevalence of bottle-feeding. An increasing level of education was significantly associated with higher levels of bottle-feeding. Mothers from communities with high education were also more likely to practice bottle-feeding. This finding was in agreement with studies conducted in Ethiopia [23], Namibia [21], and Indonesia [18]. This reflects that being educated could not guarantee increased awareness about the advantages of appropriate child feeding practices, including breastfeeding. Highly educated mothers are more likely to be employed, stop breastfeeding early, and encourage bottle use for the sake of returning to work. Therefore, the ignorance of educated women about nutrition counseling should be discouraged. Unmarried women were more likely to practice bottle-feeding than married women. This finding aligns with a study conducted in the United Kingdom [32]. This might be due to the lack of support from her partner. Lack of enough family support was one of the reasons for breastfeeding cessation [33]. Paternal involvement in baby-feeding decisions and including men in breast-feeding promotion campaigns are encouraged [34]. Non-working mothers were more likely to practice bottle-feeding compared with their counterparts. This finding was inconsistent with studies conducted in Ethiopia [24, 25, 27], Namibia [21], and Indonesia [18], in which working mothers were more likely to bottle-feed their children. This might be explained by the fact that, while it hasn’t been shown to be a barrier to breastfeeding, a mother’s work has an impact on the frequency and length of breastfeeding as well as the health of her infants [35]. Hence, designing supportive policies for non-working mothers is something to consider.

Similarly, mothers with media exposure were more likely to practice bottle-feeding. This suggests that in order to bring about significant changes in societal norms and cultural habits related to the feeding practices of infants and young children, it will be necessary to overcome literacy obstacles, provide legislative support, and provide interpersonal counseling, in addition to media [36]. Mothers from the richest households were more likely to practice bottle-feeding compared with those from the poorest households. This finding was consistent with studies conducted in Ethiopia [23], Namibia [21], and Indonesia [18]. This might be due to the fact that mothers from the richest households may have easy access to costly breastfeeding choices, which might contribute to practicing bottle feeding. According to this study, female-headed households were more likely to practice bottle-feeding compared with male-headed households. This might be explained by maternal decision-making on child feeding choices considering infant nutritional benefits, maternal benefits, and personal and professional support, as mothers are autonomous when they take on the responsibility of leading the household. The odds of bottle-feeding were higher among mothers with 5–10 household members compared with those with 1–4 household members. This might be due to the fact that mothers with large families may not have enough time to breastfeed their children and shift to bottle-feeding. Mothers with one under-five child in the household were more likely to practice bottle-feeding compared with those who had three or more under-five children. This finding was consistent with a study conducted in Holeta town, Ethiopia [27]. This might be explained by a lack of previous experience among mothers with only one child. Lack of previous experiences with children may lead mothers to bypass efforts to breastfeed and to favor bottle-feeding. Mothers who gave birth at home were more likely to practice bottle-feeding. This finding was supported by a study conducted in Asella town, Ethiopia [25]. However, this was in contrast to a study conducted in Namibia [21], in which mothers who delivered in a hospital had a higher risk of bottle-feeding. The lower odds of bottle feeding among mothers who delivered at health facilities in the current study might be due to the fact that mothers who gave birth at health facilities are more likely to get information on breastfeeding and the avoidance of bottle feeding from healthcare professionals. This implies that health facility delivery should be encouraged to improve child feeding practices.

Mode of delivery was another predictor of bottle feeding, in which cesarean section delivery increases the risk of bottle feeding. This finding was in line with a study conducted in Indonesia [18]. A cesarean section can delay the initiation of breastfeeding and shorten the duration of exclusive breastfeeding, which triggers mothers to prefer bottle feeding [37]. The perception of mothers who delivered through a cesarean section about the inadequacy of breast milk might contribute to the use of bottle feeding [38]. Mothers who were counseled on breastfeeding were more likely to practice bottle-feeding compared with their counterparts. This finding was consistent with a study conducted in Saudi Arabia [39]. Therefore, targeted interventions are required to raise mothers’ self-efficacy and motivate public health experts to change the emphasis of health promotion programs from “need to breastfeed” to “how to breastfeed.” It is advised that breastfeeding women receive counseling that is specific to their needs and the needs of the community. Children aged 6–11 months were more likely to receive bottle-feeding compared with those aged 0–5 months. This finding was in agreement with studies conducted in Ethiopia [23], Namibia [21], and Indonesia [18]. Children may have feeding alternatives as they get older, which may increase the rate of receiving bottle feeding. The higher odds of bottle feeding among children aged > 6 months might be attributed to the fact that bottle use is associated with the intake of processed milk, water, and tea, which are commonly used as the child’s age increases [40]. Mothers from urban areas were more likely to practice bottle-feeding. This finding was in line with studies conducted in Ethiopia [23], Sudan [19], Namibia [21], and Indonesia [18]. This might be due to the fact that mothers from urban areas are more likely to be from families with a higher wealth quantile, which might facilitate their access to breast-milk alternatives and information on breast-milk alternatives. This could also be explained by the greater accessibility of infant formulas at drug stores and the routine promotion of these products by pharmaceutical companies through the media in urban areas [41]. The higher odds of bottle-feeding among mothers from communities with high media exposure in the current study could also support this finding. The work conditions of mothers of children in urban areas could also contribute to encouraging them to practice bottle-feeding [42]. Hence, targeted interventions are needed among mothers who reside in rural areas to improve their child-feeding practices.

### Strengths and limitations of the study

The present study has the following strengths: First, a large sample size with weighted nationally representative data from 20 SSA countries was used. Second, a multilevel mixed-effects analysis was employed to accommodate the hierarchical nature of the DHS data and get a reliable estimate. Third, policymakers and program managers could use the findings of this study as input to design appropriate intervention strategies to improve child health, as pooled countrywide survey data is used. This study also has limitations. Firstly, the findings of the present study might be influenced by social desirability and recall biases, as the DHS survey was based on mothers’ self-reports. Secondly, the cause-and-effect relationship of variables couldn’t be established due to the cross-section nature of the data. In addition, the drawback of the secondary nature of the data is also predictable.

## Conclusion

Nearly one out of seven children aged 0 to 23 months received bottle-feeding in sub-Saharan African countries. Older mothers, higher mothers’ educational status, unmarried women, richest families, non-working mothers, exposure to media, female-headed households, having 5–10 family members, having one under-five child, home delivery, cesarean delivery, children aged 6–11 months, and urban residence were significantly associated with an increased risk of bottle feeding. Therefore, breastfeeding promotion programs are advised to target mothers who are older, educated, working, rich, gave birth at home, have a large family size, are delivered by cesarean section, have children aged 6–11 months, and reside in urban areas to achieve a significant decrease in the bottle feeding rate in sub-Saharan Africa. Further research is needed to explore the barriers to breastfeeding practice, and focus on interventions to improve breastfeeding practice among sub-Saharan African mothers.

### Electronic supplementary material

Below is the link to the electronic supplementary material.

Supplementary Material 1

## Data Availability

The data is publicly available online at https://dhsprogram.com/data/available-datasets.cfm

## Abbreviations

AOR: Adjusted Odds Ratio
CI: Confidence Interval
DHS: Demographic and Health Survey
ICC: Intra-class Correlation Coefficient
IYCF: Infant and Young Child Feeding
MOR: Median Odds Ratio
PCV: Proportional Change in Variance
PNC: Postnatal Care
SSA: sub-Saharan Africa
VIF: Variance Inflation Factor
WHO: World Health Organization
