# Supplementary material for: Bottle-feeding practice and its associated factors among mothers of children aged 0 to 23 months in sub-Saharan Africa: a multi-level analysis of demographic and health surveys (2015–2022)
Source: BMC Public Health. 2024 Jun 26;24:1712. doi: 10.1186/s12889-024-19244-9 (PMC11209972; doi:10.1186/s12889-024-19244-9)
Supplement: Supplementary file 1 — Supplementary Material 1 [file 12889_2024_19244_MOESM1_ESM.docx]

**Multicollinearity test results**

| **Variable** | **VIF** | **1/VIF** |
| --- | --- | --- |
| Wealth index | 1.59 | 0.627076 |
| Family size | 1.49 | 0.6717 |
| Educational status | 1.46 | 0.683283 |
| Number of under five children | 1.45 | 0.691751 |
| Residence | 1.38 | 0.723402 |
| Current marital status | 1.31 | 0.762924 |
| Counseling on breastfeeding | 1.30 | 0.767797 |
| Community poverty level | 1.29 | 0.774483 |
| Place of delivery | 1.28 | 0.783175 |
| Media exposure | 1.23 | 0.811113 |
| Community-level education | 1.20 | 0.835633 |
| Sex of the household head | 1.20 | 0.836251 |
| Community-level media exposure | 1.18 | 0.850613 |
| PNC checkup | 1.15 | 0.871094 |
| Maternal age | 1.14 | 0.874415 |
| Pregnancy intention | 1.07 | 0.934476 |
| Mode of delivery | 1.06 | 0.946473 |
| Maternal occupation | 1.05 | 0.954509 |
| Age of the child | 1.02 | 0.979964 |
| Sex of the child | 1.00 | 0.999485 |
| Mean VIF | 1.24 | |
